# *Toxocara canis* Infection Alters lncRNA and mRNA Expression Profiles of Dog Bone Marrow

**DOI:** 10.3389/fcell.2021.688128

**Published:** 2021-06-30

**Authors:** Wen-Bin Zheng, Yang Zou, Qing Liu, Min-Hua Hu, Hany M. Elsheikha, Xing-Quan Zhu

**Affiliations:** ^1^College of Veterinary Medicine, Shanxi Agricultural University, Jinzhong, China; ^2^State Key Laboratory of Veterinary Etiological Biology, Key Laboratory of Veterinary Parasitology of Gansu Province, Lanzhou Veterinary Research Institute, Chinese Academy of Agricultural Sciences, Lanzhou, China; ^3^National Canine Laboratory Animal Resource Center, Guangzhou General Pharmaceutical Research Institute Co., Ltd, Guangzhou, China; ^4^Faculty of Medicine and Health Sciences, School of Veterinary Medicine and Science, University of Nottingham, Loughborough, United Kingdom; ^5^Key Laboratory of Veterinary Public Health of Higher Education of Yunnan Province, College of Veterinary Medicine, Yunnan Agricultural University, Kunming, China

**Keywords:** *Toxocara canis*, toxocariasis, transcriptomics, Beagle dog, bone marrow

## Abstract

Bone marrow is the main hematopoietic organ that produces red blood cells, granulocytes, monocyte/macrophages, megakaryocytes, lymphocytes, and myeloid dendritic cells. Many of these cells play roles in the pathogenesis of *Toxocara canis* infection, and understanding how infection alters the dynamics of transcription regulation in bone marrow is therefore critical for deciphering the global changes in the dog transcriptional signatures during *T. canis* infection. In this study, long non-coding RNA (lncRNA) and messenger RNA (mRNA) expression profiles in the bone marrow of Beagle dogs infected with *T. canis* were determined at 12 h post-infection (hpi), 24 hpi, 96 hpi, and 36 days post-infection (dpi). RNA-sequencing and bioinformatics analysis identified 1,098, 984, 1,120, and 1,305 differentially expressed lncRNAs (DElncRNAs), and 196, 253, 223, and 328 differentially expressed mRNAs (DEmRNAs) at 12 h, 24 h, 96 h, and 36 days after infection, respectively. We also identified 29, 36, 38, and 68 DEmRNAs potentially *cis*-regulated by 44, 44, 51, and 80 DElncRNAs at 12 hpi, 24 hpi, 96 hpi, and 36 dpi, respectively. To validate the sequencing findings, qRT-PCR was performed on 10 randomly selected transcripts. Many altered genes were involved in the differentiation of bone marrow cells. GO of DElncRNAs and GO and KEGG pathway analyses of DEmRNAs revealed alterations in several signaling pathways, including pathways involved in energy metabolism, amino acid biosynthesis and metabolism, Wnt signaling pathway, Huntington's disease, HIF-1 signaling pathway, cGMP–PKG signaling pathway, dilated cardiomyopathy, and adrenergic signaling in cardiomyocytes. These findings revealed that bone marrow of *T. canis*-infected dogs exhibits distinct lncRNA and mRNA expression patterns compared to healthy control dogs. Our data provide novel insights into *T. canis* interaction with the definitive host and shed light on the significance of the non-coding portion of the dog genome in the pathogenesis of toxocariasis.

## Introduction

Toxocariasis, caused by *Toxocara canis* infection, is a significant public health problem in many parts of the world. Dogs serve as the definitive host for *T. canis*, where they harbor the adult nematodes in their intestine, which shed eggs in the feces. Humans are accidentally infected when they ingest food or water contaminated with *T. canis* eggs, where larvae hatch and migrate throughout the body, leading to various inflammatory conditions (e.g., visceral, ocular, and neural larva migrans) without developing into adult worms (Strube et al., 2013; Chen et al., 2018; Rostami et al., 2019a). The global prevalence of *T. canis* infection in humans and dog is 19 and 11.1%, respectively (Rostami et al., 2019b, 2020). Infection with this parasite is more widespread in socioeconomically disadvantaged populations, especially in tropical and subtropical regions (Ma et al., 2018). Toxocariasis is listed among the six Neglected Parasitic Infections targeted by the United States Centers for Disease Control and Prevention for public health action (https://www.cdc.gov/parasites/npi/).

Due to their self-renewal capacity, bone marrow-derived hematopoietic stem cells (HSCs) can differentiate into different cell types, including erythrocytes, granulocytes (neutrophils, eosinophils, and basophils), monocyte/macrophages, megakaryocytes/platelets, lymphocytes, and myeloid dendritic cells (Song et al., 2016); many of these contribute to the immune response against *T. canis* and can thus play important roles in the pathogenesis of toxocariasis. For example, eosinophils alone (Klion and Nutman, 2004) or in conjunction with other innate immune cells, such as neutrophils (Galioto et al., 2006), play a critical role in controlling helminth infection by producing toxic granule proteins and/or reactive oxygen intermediates, which damage the cuticle of the worms. Eosinophilia is a common feature of the host's response to helminth infection (Huang and Appleton, 2016) and has been detected in puppies infected by *T. canis* at 12 h post-infection (hpi), 24 hpi, 10 days post-infection (dpi), and 36 dpi (Zheng et al., 2019).

Previous genomics, transcriptomics, and proteomics studies on *T. canis* have improved the understanding of the genetic constitution and pathophysiology of *T. canis* infection (Ma et al., 2018; Zheng W. B. et al., 2020). Differences in neurotoxocarosis caused by *T. canis* and *T. cati* have been investigated by microarray analysis (Janecek et al., 2015). Additionally, metabolomics of serum and transcriptomics of lung tissue revealed several metabolic pathways and a considerable number of coding genes and long non-coding RNAs (lncRNAs), which mediate the interaction between *T. canis* and its canine host (Zheng et al., 2019, 2021). Despite these efforts, our understanding of the temporal changes of lncRNA, which are important transcriptional regulators of the immune and inflammatory responses to infection (Wang et al., 2015), in the bone marrow in dogs during *T. canis* infection is unknown.

In the present study, we investigated the changes in the global expression of lncRNAs and messenger RNAs (mRNAs) of bone marrow in Beagle dogs infected by *T. canis*. This comprehensive transcriptional analysis identified gene expression trajectories and novel mRNAs and lncRNAs that may play roles in the pathogenesis of toxocariasis.

## Materials and Methods

### Animals and Sample Collection

A total of 24 Beagle dogs, 6–7 weeks old, were purchased from and housed at the National Canine Laboratory Animal Resource Center (Guangzhou, China). The dogs did not receive any vaccination or medication before or during the experiment. They were quarantined for 1 week prior to initiation of this study for general health observations. Dogs were divided into four groups corresponding to four different time-points post-infection: 12 hpi, 24 hpi, 96 hpi, and 36 dpi. Complete blood examination using automated blood analyzer (XT2000 iv; Sysmex, Kobe, Japan) and fecal examination using a sugar floating method were performed to ensure that all puppies are free of any intestinal parasitic infection prior to the study. Puppies from the same litters were allocated randomly by a researcher unaware of experimental design into infected group and control group, with three biological replicates in each group to reduce background differences. Puppies of the infected groups were infected orally with 1 ml of saline solution containing 300 infectious *T. canis* eggs, while puppies of the control groups were mock-infected orally with 1 ml saline only. At each of the above indicated time points post-infection, puppies from one infected group and a matched control group were euthanized by injecting KCl into the heart under general anesthesia using Zoletil 50 (Virbac, Nice, France). Then, the tibia of each puppy was rapidly separated, and the tibial bone marrow was collected under sterile conditions and stored in liquid nitrogen for RNA extraction. The liver and half of the lungs of each puppy were shredded to recover *T. canis* larvae, as described previously (Zheng et al., 2019). Blood profiling of each puppy was performed using automated blood analyzer to count the number of red blood cells, white blood cells, neutrophils, and eosinophils. The potential laboratory waste and infectious clinical waste, such as the infectious eggs and remaining puppy's tissues, were decontaminated by autoclaving prior to disposal.

### RNA Extraction and Transcriptome Sequencing (RNA-seq) Analysis

Total RNA of each bone marrow was extracted using TRIZOL (Life Technologies, Carlsbad, USA), and genomic DNA was removed from total RNA using DNase I (NEB, Ipswich, USA). The ribosomal RNA was depleted from the total RNA using the Epicentre Ribo-zeroTM rRNA Removal Kit (Madison, WI, USA), and rRNA-free residue was cleaned up by ethanol precipitation. Then, 24 cDNA sequencing libraries were generated and RNA-Seq were performed on the Illumina NovaSeq platform and 150 bp paired-end reads were generated.

### Identification of lncRNAs and Differentially Expressed lncRNAs and mRNAs

Clean reads were obtained by removing reads with poly-N, adapters, insert tags, and low quality reads and were mapped to the reference genome of *Canis lupus familiaris* using HISAT2 (v2.0.4) (Langmead and Salzberg, 2012). Genome and annotation files of *Canis lupus familiaris* were downloaded from the Ensembl database (CanFam3.1), and the mapped reads were aligned and assembled using StringTie (v1.3.3) (Pertea et al., 2016). The sequences of the coding genes were quantified by HTSeq (v0.11.4) (Anders et al., 2015). The fragments per kilobase of exon model per million mapped reads (FPKM) was used for transcripts analysis. Transcripts with exon ≥2 and length >200 bp were used to identify lncRNA. Transcripts that have coding potential were filtered out by using CNCI (coding-non-coding index), CPC2 (coding potential calculator), and PFAM protein domain analysis (Punta et al., 2012; Sun et al., 2013; Kang et al., 2017), and the rest of transcripts without coding potential were considered as novel lncRNAs. Differential expression analysis of lncRNA and mRNA between each infected and control group was performed using the bioconductor package DESeq2 (Love et al., 2014). A significance threshold of |log2 (fold change)| ≥1 and *P* < 0.05 was used to identify differentially expressed genes.

### Target Gene Prediction of lncRNAs and Functional Analysis of lncRNAs and mRNAs

The *cis* target gene, located within the upstream or downstream 100 kb of lncRNAs, was analyzed to predict the lncRNA function. To investigate the functions of the differentially expressed lncRNAs (DElncRNAs) and differentially expressed mRNAs (DEmRNAs) in puppies' bone narrow, GOseq R package (Young et al., 2010) was used to perform Gene Ontology (GO) analysis based on the *cis* target gene of DElncRNAs and DEmRNAs. GO terms were classified into three categories, including biological process (BP), cellular component (CC), and molecular function (MF). In addition, KOBAS (Wu et al., 2006) was used to map genes and to identify KEGG signaling pathways based on the DEmRNAs. GO terms and KEGG pathways with *P* < 0.05 were considered significantly enriched. The relationship between DElncRNAs and its *cis* DEmRNAs was visualized by Cytoscape v.3.5 (Lotia et al., 2013).

### Quantitative RT-PCR Validation of RNA-seq Results

Ten transcripts (five DEmRNAs and five DElncRNAs) at each of the four infection stages were randomly selected to validate the RNA-Seq results by quantitative real-time PCR (qRT-PCR) on LightCycler480 (Roche, Basel, Switzerland). Commercial kits, amplification conditions, and melting curve analysis for mRNA and lncRNA amplification were performed as described previously (Zheng et al., 2021). The expression levels of the selected DEmRNAs and DElncRNAs were normalized to that of the house-keeping genes. All genes and primers are shown in Supplementary Table 1.

## Results

### Confirmation of *T. canis* Infection

**Figure 1:**
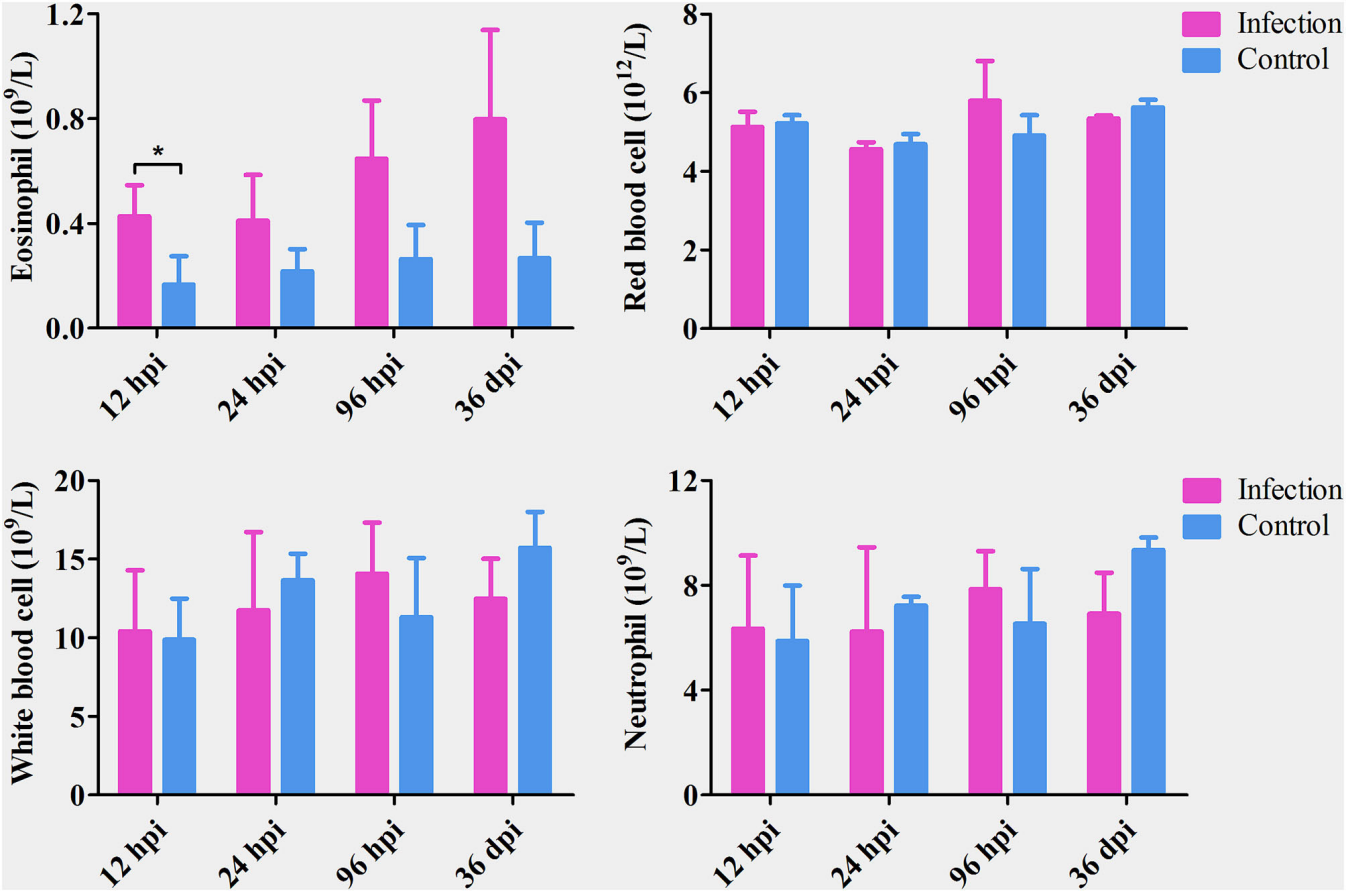
The number of eosinophils, red blood cells, white blood cells, and neutrophils in the blood of *Toxocara canis*-infected (infection) and uninfected (control) groups of Beagle dogs at the indicated time points post-infection. The graphs represent the means ± standard deviations of the results obtained from three dogs per group. Statistical analysis was performed using *t*-test using GraphPad Prism 5 software. Asterisk denotes significance (*P < 0.05).

The number of eosinophils increased gradually from 12 hpi to 36 dpi; however, the difference between infected and control dogs was statistically significant only at 12 hpi (*P* < 0.05). There was no significant difference in the number of red blood cells, white blood cells, or neutrophils between infected and control dogs at any time point after infection (Figure 1). At 12 and 24 hpi, *T. canis* larvae were found in the livers of all infected puppies with an average of 1.33 and 9 larvae per liver, respectively. At 96 hpi, *T. canis* larvae were found in livers and lungs of all infected puppies with an average of 21 and 27.3 larvae per liver and lung, respectively. At 36 dpi, only two larvae were found in one liver, and an average of 75.3 *T. canis* adult roundworm were found in small intestines of all infected puppies (Table 1). As expected, no *T. canis* was found in puppies in the control groups. These results are consistent with those reported previously (Zheng et al., 2021).

**Table 1:** The recovery of *Toxocara canis* larvae from the liver, lung, and small intestine of infected puppies at four time points post-oral infection with 300 infectious *T. canis* eggs.

**Time after infection**	**Liver**			**Lung**			**Small intestine**		
	**No. examined**	**No. positive**	Mean^*^	**No. examined**	**No. positive**	Mean^*^	**No. examined**	**No. positive**	Mean^*^
12 hpi	3	3	1.33	3	0	0	3	0	0
24 hpi	3	3	9	3	0	0	3	0	0
96 hpi	3	3	21	3	3	27.3	3	0	0
36 dpi	3	1	0.67	3	0	0	3	3	75.3

* *The average number of T. canis larvae recovered from each organ of the examined dogs*.

### Overview of RNA-seq and Differential Expression of lncRNAs and mRNAs

**Figure 2:**
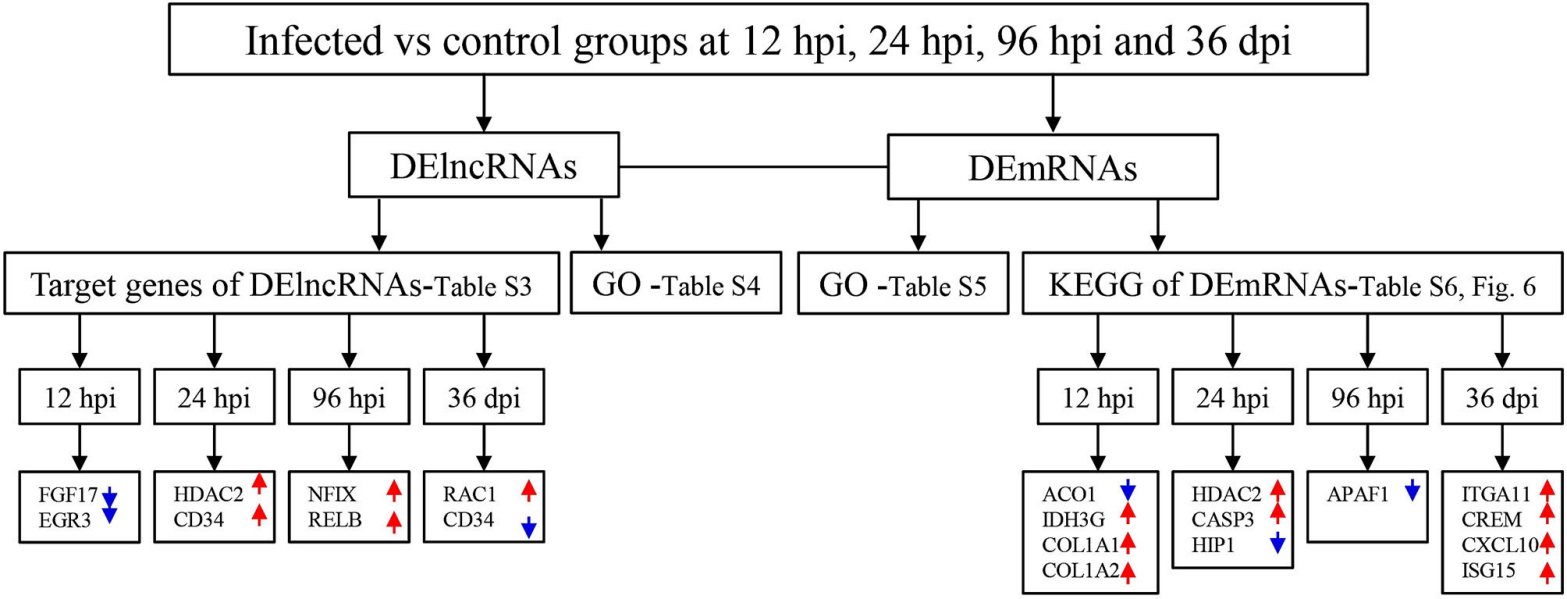
The workflow of data processing. First, long non-coding RNAs (lncRNAs) and mRNAs were obtained by sequencing and the differentially expressed (DE) transcripts of lncRNAs and mRNAs were determined in the bone marrow of Beagle dogs at different stages of *Toxocara canis* infection. Then, Gene Ontology (GO) terms of the target genes of DElncRNAs, as well as GO terms and Kyoto Encyclopedia of Genes and Genomes (KEGG) pathway of DEmRNAs were determined. Increased and decreased expression of the transcripts are indicated by red upward pointing arrows and blue downward pointing arrows, respectively.

**Figure 3:**
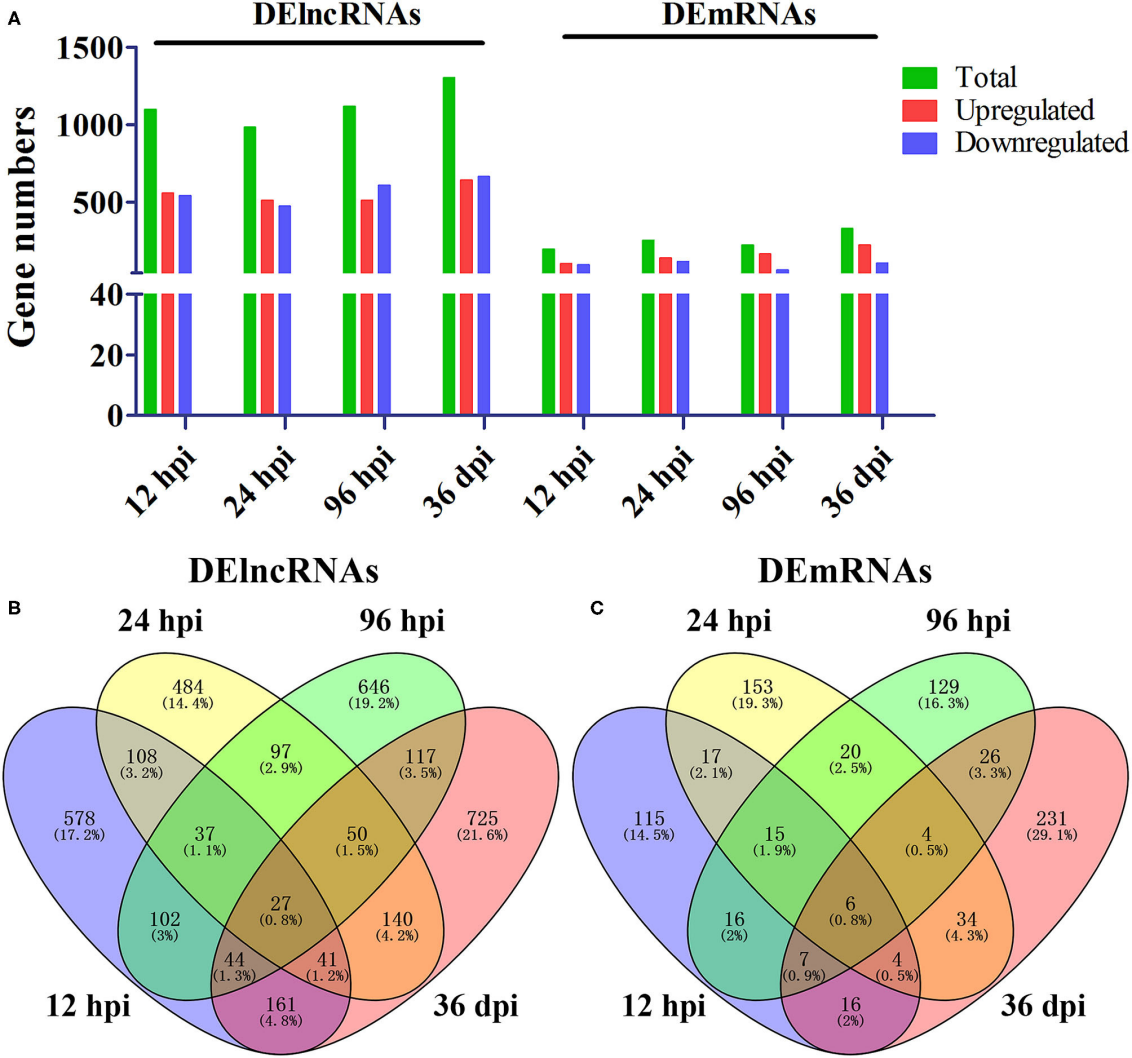
Comparisons of differentially expressed lncRNAs and mRNAs in the bone marrow of Beagle dogs infected by *Toxocara canis* at 12 h post-infection (hpi), 24 hpi, 96 hpi, and 36 day post-infection (dpi). **(A)** The number of DElncRNAs and DEmRNAs at four infection stages. Green, red, and blue colors represent the number of total, upregulated, and downregulated transcripts, respectively. **(B,C)** Venn diagrams showing the common and unique DElncRNAs and DEmRNAs at the four indicated time points after infection between infected and control groups.

**Figure 4:**
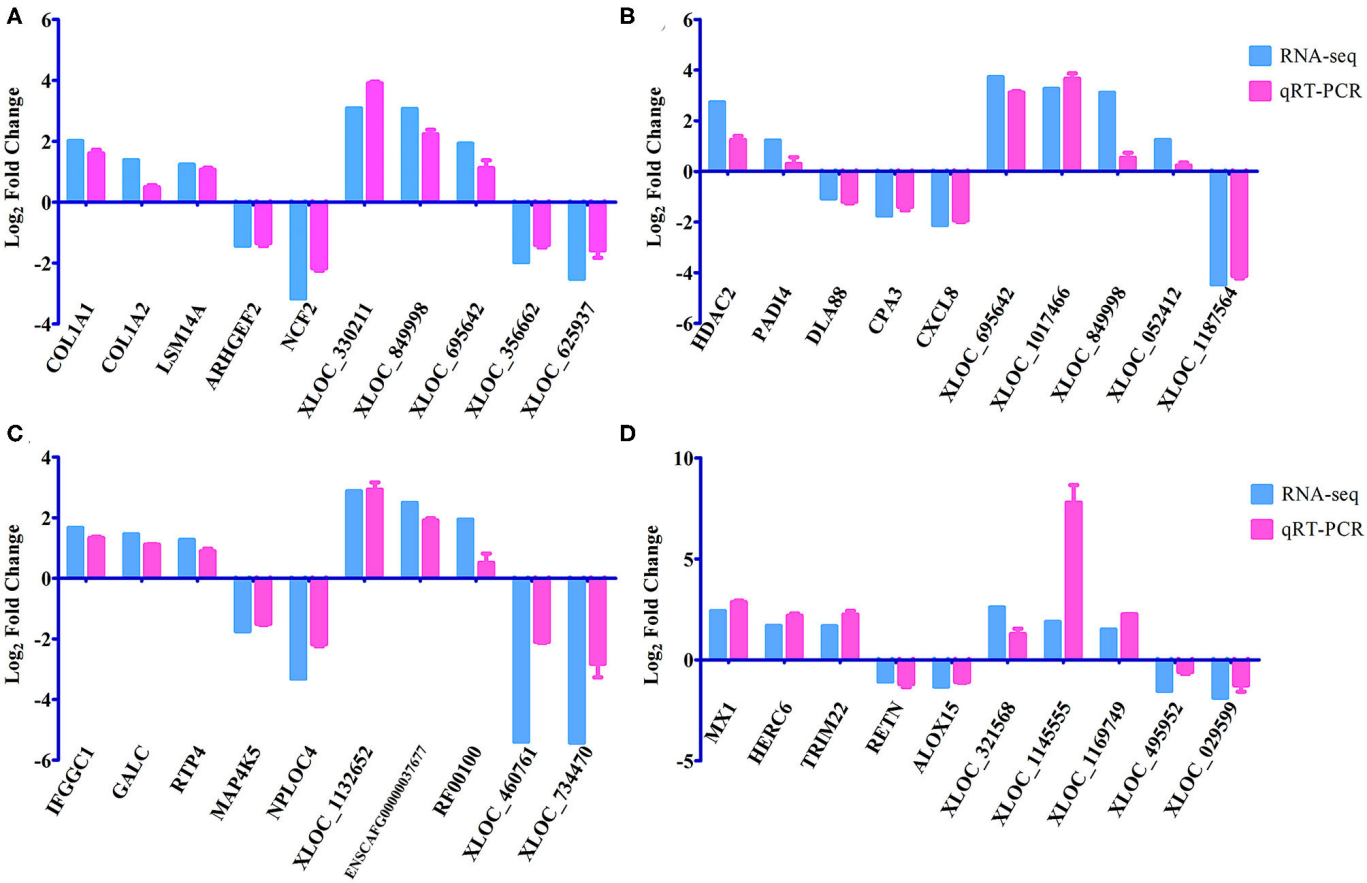
Verification of the expression of DEmRNAs and DElncRNAs using qRT-PCR at **(A)** 12 hpi, **(B)** 24 hpi, **(C)** 96 hpi, and **(D)** 36 dpi. The Y-axis denotes the log_2_ fold change, and the X-axis shows the analyzed DEmRNAs and DElncRNAs. The error bars represent the standard deviation based on three replicates.

A total of 24 puppies bone marrow were used for RNA-seq, generating 3,378,382,852 raw reads and 3,297,334,736 clean reads, with an average of 20.61 Gb clean data from each sample. A total of 1,272 annotated lncRNA transcripts, 24,794 novel lncRNA transcripts, and 25,157 mRNA transcripts were identified. The workflow of data processing and the associated outputs are shown in Figure 2. At 12 hpi, 1,098 DElncRNAs and 196 DEmRNAs were identified between infected and uninfected groups. At 24 hpi, 984 DElncRNAs and 253 DEmRNAs were identified. At 96 hpi, 1,120 DElncRNAs and 223 DEmRNAs were identified. At 36 dpi, 1,305 DElncRNAs and 328 DEmRNAs were detected (Figure 3; Supplementary Table 2). The RNA-seq results were validated using five DEmRNAs and five DElncRNAs at each infection stage using qRT-PCR analysis (Figures 4A–D). The qRT-PCR results showed an overall consistency in the trend and magnitude of the expression obtained by RNA-seq analysis.

### Target Gene Prediction and GO Annotation Analysis of lncRNAs

A total of 22,736 mRNA transcripts were found within the upstream and downstream 100 kb of the 22,393 lncRNA transcripts. At 12 hpi, 29 DEmRNA transcripts (e.g., *fgf17* and *egr3*) were found within the upstream and downstream of 44 DElncRNA transcripts, forming 45 pairs. At 24 hpi, 36 DEmRNA transcripts (e.g., *hdac2* and *cd34*) were found within the upstream and downstream of 44 DElncRNA transcripts, forming 49 pairs. At 96 hpi, 38 DEmRNA transcripts (e.g., *nfix* and *relb*) were found within the upstream and downstream of 51 DElncRNA transcripts, forming 53 pairs. At 36 hpi, 68 DEmRNA transcripts (e.g., *rac1* and *cd34*) were found within the upstream and downstream of 80 DElncRNA transcripts, forming 91 pairs (Supplementary Figure 1; Supplementary Table 3). GO annotation analysis was performed using target genes of DElncRNAs, showing that 2,926 target genes were significantly enriched in 681 GO terms at 12 hpi, including regulation of defense response, protein–DNA complex, and regulation of response to stress; 2,623 target genes were significantly enriched in 688 GO terms at 24 hpi, including double-stranded RNA binding, nucleoplasm, and nuclear part; 3,023 target genes were significantly enriched in 676 GO terms at 96 hpi, including intracellular membrane-bounded organelle, intracellular part, and membrane-bounded organelle; 3,268 target genes were significantly enriched in 650 GO at 36 dpi, including immunoglobulin complex, complement activation, classical pathway, and endomembrane system (Supplementary Table 4). The top 30 differential GO terms of DElncRNA target genes are shown in Supplementary Figure 2.

### GO and KEGG Pathway Analysis of Differentially Expressed mRNAs

**Figure 5:**
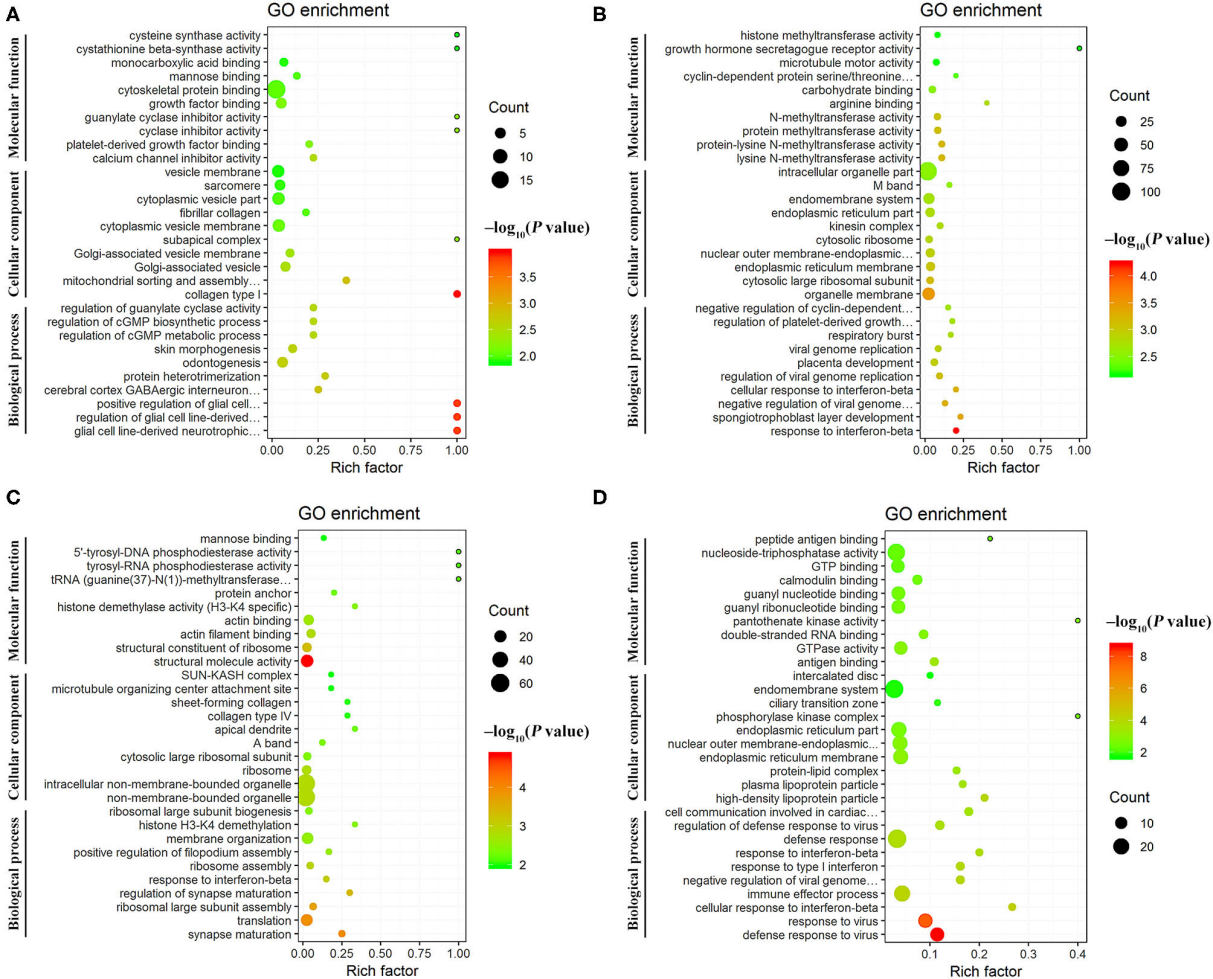
Scatter plots of the top 30 enriched Gene Ontology (GO) terms (including biological process, cellular component, and molecular function categories) of the DEmRNAs at **(A)** 12 hpi, **(B)** 24 hpi, **(C)** 96 hpi, and **(D)** 36 dpi. The X-axis label represents the rich factor; the Y-axis label shows the GO terms. The rich factor reflects the proportion of DEmRNAs in a given GO term. The greater the rich factor, the greater the degree of enrichment. The color of the dots represents the enrichment score [–log_10_(*P*-value)], where red color indicates high enrichment, while green color indicates low enrichment. Dot size represents the number of DEmRNAs in the corresponding GO term (bigger dots indicate larger DEmRNA numbers).

GO annotation analysis identified 126 DEmRNAs that were significantly enriched in 358 GO terms at 12 hpi, including 270 BP terms, 36 CC terms, and 52 MF terms, including collagen type I, glial cell line-derived neurotrophic factor secretion, and mitochondrial sorting and assembly machinery complex. At 24 hpi, 189 DEmRNAs were significantly enriched in 415 GO terms, including 295 BP terms, 53 CC terms, and 67 MF terms, including response to interferon-beta, organelle membrane, and spongiotrophoblast layer development. At 96 hpi, 162 DEmRNAs were significantly enriched in 232 GO terms, including 142 BP terms, 32 CC terms, and 58 MF terms, including structural molecule activity, synapse maturation, and ribosomal large subunit assembly. At 36 dpi, 222 DEmRNAs were significantly enriched in 297 GO, including 210 BP terms, 22 CC terms, and 65 MF terms, including defense response to virus, cellular response to interferon-beta, and immune effector process (Supplementary Table 5). The top 30 differential GO terms are shown in Figure 5.

**Figure 6:**
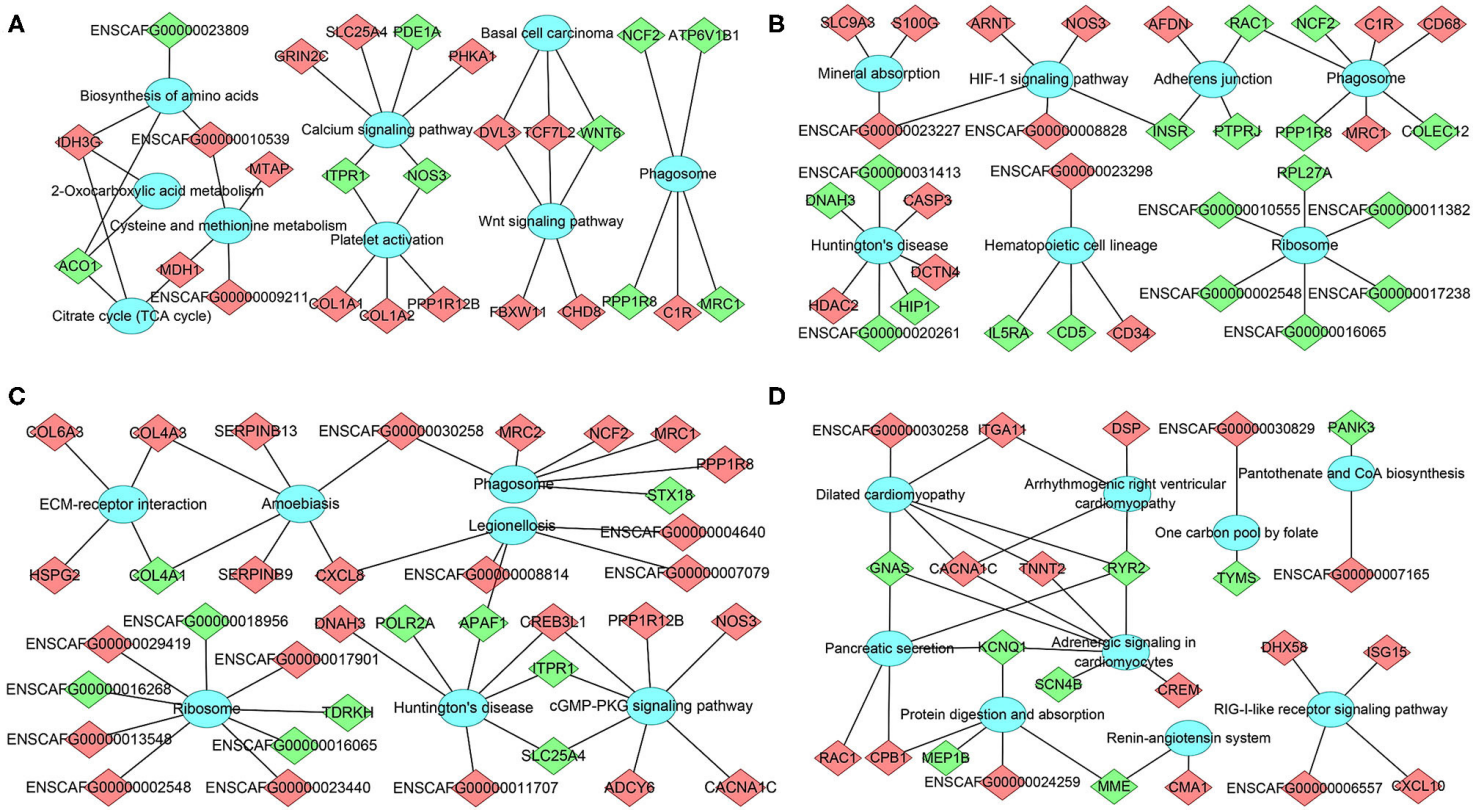
The network of DEmRNA-pathway using KEGG analysis at **(A)** 12 hpi, **(B)** 24 hpi, **(C)** 96 hpi, and **(D)** 36 dpi. Diamonds represent the DEmRNA, and ellipses represent the significantly enriched pathways. Red color indicates upregulation, while green color indicates downregulation.

The mRNA-pathway network was constructed to identify the DEmRNAs that connect the pathways (Figure 6). These results showed that 26 DEmRNAs (e.g., *col1a1, col1a2*, and *nos3*) were significantly enriched in 9 pathways at 12 hpi, including calcium signaling pathway, platelet activation, and Wnt signaling pathway; 33 DEmRNAs (e.g., *casp3, hip1*, and *insr*) were significantly enriched in seven pathways at 24 hpi, including phagosome, Huntington's disease, and hematopoietic cell lineage; 36 DEmRNAs (e.g., *apaf1, c1r*, and *ncf2*) were significantly enriched in seven pathways at 96 hpi, including ribosome, legionellosis, and cGMP–PKG signaling pathway; 24 DEmRNAs (e.g., *itga11, crem*, and *gnas*) were significantly enriched in nine pathways at 36 dpi, including protein digestion and absorption, pancreatic secretion, and RIG-I-like receptor signaling pathway (Supplementary Table 6). The top 20 most highly represented pathways in each group are shown in Supplementary Figure 3.

## Discussion

We have generated a new resource of transcriptional signatures from the bone marrow of dogs during *T. canis* infection to improve the understanding of the pathogenesis for toxocariasis. We determined the lncRNA and mRNA expression patterns at 12 hpi, 24 hpi, 96 hpi, and 36 dpi using RNA-seq analysis. At 12 hpi, the transcripts of fibroblast growth factor 17 (*fgf17*) were significantly decreased to 0. The *fgf17* gene plays a role in the brain development, and its deficiency causes abnormal social behaviors in mice (Ford-Perriss et al., 2001; Scearce-Levie et al., 2008). *FGF17* is also an autocrine prostatic epithelial growth factor and plays a role in human prostate carcinogenesis (Polnaszek et al., 2004). In this study, three upregulated and three downregulated lncRNA transcripts that are located in the vicinity of *fgf17* were identified, suggesting that in the bone marrow, *fgf17* and its related lncRNAs may play roles during the early stage of *T. canis* infection. Early growth response gene 3 (*egr3*), a member of zinc-finger transcription factors family, is a key negative regulator of T cell activation, and its overexpression inhibits transcription of IL-2 promoter (Safford et al., 2005). Also, *egr3* is a potent inhibitor of HSCs (Cheng et al., 2015). The transcription level of *egr3* was significantly decreased to 0 at 12 hpi. The downregulation of *egr3* may lead to the activation of common lymphoid and myeloid progenitors. Moreover, the level of XLOC_596134, which is located in the vicinity of the *egr3* gene, was upregulated 18.4 times.

At 24 hpi, the transcription level of histone deacetylase 2 (*hdac2*) was upregulated 6.61 times. This gene plays a role in HSC's homeostasis (Heideman et al., 2014). Simultaneous loss of *hdac1* and *hdac2* genes results in the depletion of HSCs and early hematopoietic progenitors, leading to anemia and thrombocytopenia (Wilting et al., 2010; Heideman et al., 2014). In the present study, the transcription level of *hdac1* was not significantly altered; however, *hdac2* was significantly upregulated, which may contribute to self-renewal of HSCs and hematopoietic differentiation at 24 hpi. In addition, the level of XLOC_159843, which is located in the vicinity of *hdac2*, was downregulated 49.74 times, which may contribute to the transcription of *hdac2*. CD34 is a well-known marker of hematopoietic stem/progenitor cells (HSPCs), and its expression is typically lost as these cells mature into terminal effectors (Hughes et al., 2020). Eosinophils arise from eosinophil-committed CD34^+^ bone marrow progenitors (pEo) (Robida et al., 2018). Mature eosinophils lose CD34 during terminal differentiation under homeostatic conditions; however, during inflammatory responses, CD34^+^ progenitors expand in the bone marrow and are released into circulation (Rådinger et al., 2011). In this study, the transcription level of CD34 was upregulated, and XLOC_1055137, which is located in the vicinity of CD34, was downregulated at 12 hpi and 24 hpi, which may play a role in the induction of eosinophilia in the peripheral blood of infected puppies during early infection.

At 96 hpi, the upregulation of Nuclear factor I/X (*NFIX*) transcription may be affected by the downregulation of XLOC_471504 and the upregulation of XLOC_459365 located in the vicinity of the *NFIX* (Supplementary Figure 1C). *NFIX*, a member of the nuclear factor I (*NFI*) family of transcription factors, is highly expressed by HSPCs of bone marrow (Holmfeldt et al., 2013) and can influence the fate of HSPCs; loss of *nfix* expression promotes B lymphopoiesis and impairs myelopoiesis (O'Connor et al., 2015). Therefore, upregulation of *nfix* at 24 hpi may contribute to the formation of eosinophils and other granulocytes. The *relb*, an NF-κB family transcription factor, is required for bone marrow-derived dendritic cell (DC) development, and its expression level determines the role of DCs in immune responses (Wu et al., 1998) and contributes to the differentiation of various progenitors (Cejas et al., 2005). The transcription level of *relb* was upregulated 10.84 times at 96 hpi, suggesting that progenitor cells were differentiated into well-developed DCs. The downregulation of XLOC_029260 may contribute to the upregulation of *relb*.

At 36 hpi, the transcription level of Ras-related C3 botulinum toxin substrate 1 (*rac1*) was significantly upregulated. *Rac1* is a highly conserved member of the RHO family of small GTPases and plays roles in numerous cellular processes *via* downstream effectors, such as actin dynamics, differentiation, inflammatory responses, proliferation, survival, and reactive oxygen species production (Cannon et al., 2020). Hematopoietic stem and progenitor cell adhesion, maintenance, and migration can be enhanced by *rac1* activation (Nguyen et al., 2017). Therefore, the upregulation of *rac1* may play an important role in maintaining HSPC self-renewal, proliferation, and differentiation during *T. canis* infection. Different from the transcription level of CD34 at 12 hpi and 24 hpi, the transcription level CD34 was significantly downregulated at 36 dpi. The downregulation of CD34 suggests that the number of HSPCs and eosinophils was decreased in bone marrow at 36 dpi. The temporal differences in the transcription level of CD34 in the bone marrow (increase at 12 hpi and 24 hpi and decrease at 36 dpi) may suggest infection stage-specific differences in the level of eosinophilia and can be attributed to the differential expression of XLOC_1055137; however, this requires further investigation.

At 12 hpi, some altered genes (e.g., *aco1, mdh1*, and *idh3g*) were enriched in signaling pathways associated with energy metabolism, and amino acid biosynthesis and metabolism, suggesting that the bone marrow metabolism of infected puppies was altered during the early stage of *T. canis* infection. In addition, many altered genes were associated with the differentiation of bone marrow mesenchymal stem cells (MSCs) at 12 hpi, of which two downregulated genes (*itpr1* and *nos3*) and three upregulated genes (*col1a1, col1a2*, and *ppp1r12b*) were enriched in platelet activation signaling pathway. The *col1a1* and *col1a2* genes encode two α chains of the type i collagen, which is the major component of the bone matrix (Pollitt et al., 2006); three downregulated genes (*itpr1, nos3*, and *pde1a*) and three upregulated genes (*grin2c, slc25a4*, and *phka1*) were enriched in calcium signaling pathway, which contributes to the osteogenic differentiation of MSCs (Hui et al., 2019). Furthermore, Wnt signaling pathway was significantly upregulated at 12 hpi, which may enhance the differentiation and mineralization of bone marrow MSCs (Ruan et al., 2021). These results suggest that metabolic alterations and MSCs differentiation in the bone marrow may be involved in the pathogenesis of *T. canis* infection at 12 hpi.

At 24 hpi, 189 DEmRNAs were significantly enriched in 415 GO terms (Supplementary Table 5) and 33 DEmRNAs were significantly enriched in 7 KEGG signaling pathways at 24 hpi (Figure 6B). *Hdac2* belongs to Huntington's disease pathway, which was significantly altered at 24 hpi. There were three upregulated genes (e.g., *hdac2, casp3*, and *dctn4*) and four downregulated genes (e.g., *dnah3, hip1, ENSCAFG00000020261*, and *ENSCAFG00000031413*) in this signaling pathway. Caspase-3 (*casp3*) is a cysteine protease that plays a crucial role in apoptosis and inflammatory responses (Takashi et al., 2020) and osteogenic differentiation of bone marrow stromal stem cells (Miura et al., 2004). Our observation that the expression of *casp3* in the bone marrow was increased 30.49 times at 24 hpi is consistent with a previous result detected in Beagle's lungs infected *T. canis* at the same time after infection (Zheng et al., 2021), suggesting that *casp3* may play various roles during *T. canis* infection. Huntingtin interacting protein 1 (HIP1) is necessary to maintain cellularity in multiple tissue types, and the mutation of *hip1* gene in mouse can cause abnormal hematopoiesis and spinal defects (Oravecz-Wilson et al., 2004). HIP1 is also an endocytic protein and is overexpressed in a variety of human cancers. Moreover, the overexpression of *hip1* in bone marrow is associated with a lower overall survival in acute myeloid leukemia patients (Wang et al., 2017). The transcription level of *hip1* was significantly decreased 81.73 times at 24 hpi, which may contribute to limiting of the proliferation of myeloid primordial cells in the hematopoietic system and maintaining the homeostasis of the bone marrow environment during *T. canis* infection. The HIF-1 signaling pathway was another significantly enriched signaling pathway with one downregulated gene (*insr*) and four upregulated genes (*arnt, nos3*, ENSCAFG00000023227 and ENSCAFG00000008828) at 24 hpi (Figure 6B). The upregulation of HIF-1 signaling pathway can promote the metabolism and behavior of MSCs (Zhou et al., 2020). Moreover, hematopoietic cell lineage was enriched with two downregulated genes and two upregulated genes at 24 hpi (Figure 6B). These results show that many altered genes or signaling pathways associated with maturation and/or differentiation of HSPCs and MSCs are identified at 24 hpi, which provides the basis for the differentiation of HSPCs into different types of cells and changes the hematopoietic microenvironment after *T. canis* infection.

Similar to 24 hpi, Huntington's disease pathway was significantly enriched at 96 hpi with three upregulated genes (*creb3l1, dnah3*, and ENSCAFG00000011707) and four downregulated genes (*apaf1, itpr1, polr2a*, and lc25a4), among which apoptotic protease activating factor 1 (APAF1) is a key molecule in the intrinsic and mitochondrial pathway of apoptosis (Shakeri et al., 2017). Apoptosis is highly important for the homeostasis and development of the hematopoietic system, and deregulation of apoptosis leads to the development of a number of human diseases (Testa and Riccioni, 2007). The inactivation or inhibition of *apaf1* expression contributes to the development of acute myeloid leukemia (Testa and Riccioni, 2007; Rostami et al., 2017). In the present study, the expression of *apaf1* was decreased to 0 at 96 hpi, suggesting that the decrease of *apaf1* transcription level induced by *T. canis* infection may cause abnormal differentiation of HSPCs. Moreover, ECM–receptor interaction and cGMP–PKG signaling pathway were significantly upregulated at 96 hpi, which may play important roles in the homeostasis of bone marrow microenvironment. Phagosome is another pathway that seems to play roles during early *T. canis* infection, with one upregulated gene and four downregulated genes at 12 hpi, three upregulated genes and four downregulated genes at 24 hpi, as well as five upregulated genes and one downregulated gene at 96 hpi (Figure 6C), among which *c1r, mrc1/2, ncf2*, and *ppp1r8* may play roles in *T. canis*–host interaction.

At 36 dpi, dilated cardiomyopathy was the most significantly enriched pathway with four upregulated genes (*cacna1c, itga11, tnnt2*, and ENSCAFG00000030258) and two downregulated genes (*gnas* and *ryr2*). The adrenergic signaling pathway in cardiomyocytes was also highly enriched with three upregulated genes (*cacna1c, tnnt2*, and *crem*) and four downregulated genes (*gnas, ryr2, kcnq1*, and *scn4b*). Integrin alpha11 (*ITGA11*) is an osteolectin receptor, and the deletion of *itga11* in mouse and human bone marrow stromal cells impairs osteogenic differentiation and blocks their response to osteolectin (Shen et al., 2019). The cAMP responsive element modulator (*CREM*) proteins are members of the activating transcription factor/cAMP response element binding protein (ATF/CREB) transcription factor family (De Cesare and Sassone-Corsi, 2000). *Crem* knock-out mice can display slightly decreased osteoclast number and increased long bone mass (Liu et al., 2007). Guanine nucleotide binding protein subunit α (*gnas*) is a key component of the cell membrane receptor pathway, and downregulation of *gnas* can repress osteogenic differentiation of bone marrow-derived MSCs, aggravating the progression of osteoporosis (An et al., 2019; Zheng X. et al., 2020). The expression of *itga11* and *crem* was upregulated 158.10 times and 6.23 times in bone marrow at 36 dpi, respectively, while the expression of *gnas* was downregulated 86.31 times at 36 dpi, suggesting that although *T. canis* worms mainly reside in the intestine at 36 dpi, they can still influence the bone marrow, especially the host MSCs. The effects of these altered genes at 36 dpi on *T. canis*–host interaction merits further investigation.

In the RIG-I-like receptor signaling pathway, the four upregulated genes included *cxcl10, dhx58, isg15*, and ENSCAFG00000006557. CXC motif chemokine 10 (CXCL10), also known as IP-10, is a small cytokine-like protein secreted by various cell types and plays a role in chemotaxis, immunomodulation, inflammation, leukocyte trafficking, and hematopoiesis (Bagheri et al., 2020). CXCL10 plays a protective role during parasitic protozoa infection, such as *Leishmania infantum* and *Toxoplasma gondii* (Vargas-Inchaustegui et al., 2010; Norose et al., 2011). The expression of *cxcl10* was upregulated 7.32 times in bone marrow at 36 dpi; however, the role of *cxcl10* during *T. canis* infection remains unknown. Interferon-stimulated gene product 15 (ISG15) is a ubiquitin-like protein, which is critical for controlling microbial infections, especially viral infection (Perng and Lenschow, 2018). In this study, the expression of *isg15* was upregulated 3.73 times; however, its role in *T. canis*–host interaction remains to be elucidated.

## Conclusions

We have identified many dysregulated lncRNAs and mRNAs in bone marrow samples obtained from *T. canis*-infected compared to uninfected Beagle dogs. GO and KEGG pathway analyses revealed DElncRNAs and DEmRNAs involved in a number of key biological processes and pathways, which may have relevance to the pathogenesis of toxocariasis. Further investigations are required to determine the underlying biological functions of the dysregulated lncRNAs and mRNAs in the pathogenesis of toxocariasis and to test their potential utility as diagnostic biomarkers for toxocariasis.

## Data Availability Statement

The datasets supporting the findings of this article are included within the paper and its Supplementary Material. The RNA-seq raw data described in the present study has been submitted to the NCBI Short Read Archive database (https://www.ncbi.nlm.nih.gov/sra) under the bio-project number PRJNA673639.

## Ethics Statement

The animal study was reviewed and approved by The Animal Ethics Committee of the Lanzhou Veterinary Research Institute, Chinese Academy of Agricultural Sciences.

## Author Contributions

HME and X-QZ conceived the idea and designed the experiments. W-BZ and YZ performed the experiments, analyzed the data, and drafted the manuscript. QL and M-HH participated in the implementation of the study. QL, HME, and X-QZ critically revised the manuscript. All authors read and approved the final version of the manuscript.

## Conflict of Interest

M-HH was employed by the company National Canine Laboratory Animal Resource Center, Guangzhou General Pharmaceutical Research Institute Co., Ltd. The remaining authors declare that the research was conducted in the absence of any commercial or financial relationships that could be construed as a potential conflict of interest.

## Supplementary Material

Supplementary Figure 1 Co-localization between the differentially expressed lncRNAs and their predicted differentially expressed target mRNAs. Red and green colors represent upregulated and downregulated transcripts, respectively. The ellipses denote mRNAs, and diamonds denote lncRNAs. **(A–D)** Represent the co-localization relationships at 12 hpi, 24 hpi, 96 hpi, and 36 dpi, respectively.

Supplementary Figure 2 Scatter plots of the top 30 enriched Gene Ontology (GO) terms (including biological process, cellular component, and molecular function categories) of the DElncRNA target genes at **(A)** 12 hpi, **(B)** 24 hpi, **(C)** 96 hpi, and **(D)** 36 dpi. The X-axis label represents the rich factor; the Y-axis label shows the GO terms. The rich factor reflects the proportion of DEmRNAs in a given GO term. The higher the rich factor, the greater the degree of enrichment. The color of the dots represents the enrichment. Score [–log_10_(*P*-value)], where red color indicates high enrichment, while green color indicates low enrichment. Dot size represents the number of DEmRNAs in the corresponding GO term (bigger dots indicate larger DEmRNA numbers).

Supplementary Figure 3 Scatter plots of the top 20 enriched Kyoto Encyclopedia of Genes and Genomes (KEGG) pathways of the DEmRNAs at **(A)** 12 hpi, **(B)** 24 hpi, **(C)** 96 hpi, and **(D)** 36 dpi. The X-axis label represents the rich factor; the Y-axis label shows the KEGG pathways. The rich factor reflects the proportion of DEmRNAs in a given pathway. The greater the rich factor, the greater the degree of pathway enrichment. The color of the dots represents the enrichment score [–log_10_(*P-*value)], where red color indicates high enrichment, while green color indicates low enrichment. Dot size represents the number of DEmRNAs in the corresponding pathway (bigger dots indicate larger DEmRNA numbers).

Supplementary Table 1 The primers used in the qRT-PCR experiment.

Supplementary Table 2 The differentially expressed lncRNAs and mRNAs at 12 hpi, 24 hpi, 96 hpi, and 36 dpi.

Supplementary Table 3 Co-localization between the differentially expressed lncRNAs and their predicted differentially expressed *cis* target genes.

Supplementary Table 4 Gene Ontology (GO) terms of *cis* target genes of the differentially expressed lncRNAs.

Supplementary Table 5 Gene Ontology (GO) terms of the differentially expressed mRNAs.

Supplementary Table 6 Signaling pathways of the differentially expressed mRNAs.
