# Supplementary figures and images for: Toxocara canis Infection Alters lncRNA and mRNA Expression Profiles of Dog Bone Marrow
Source: Front Cell Dev Biol. 2021 Jun 30;9:688128. doi: 10.3389/fcell.2021.688128 (PMC8277978; doi:10.3389/fcell.2021.688128)

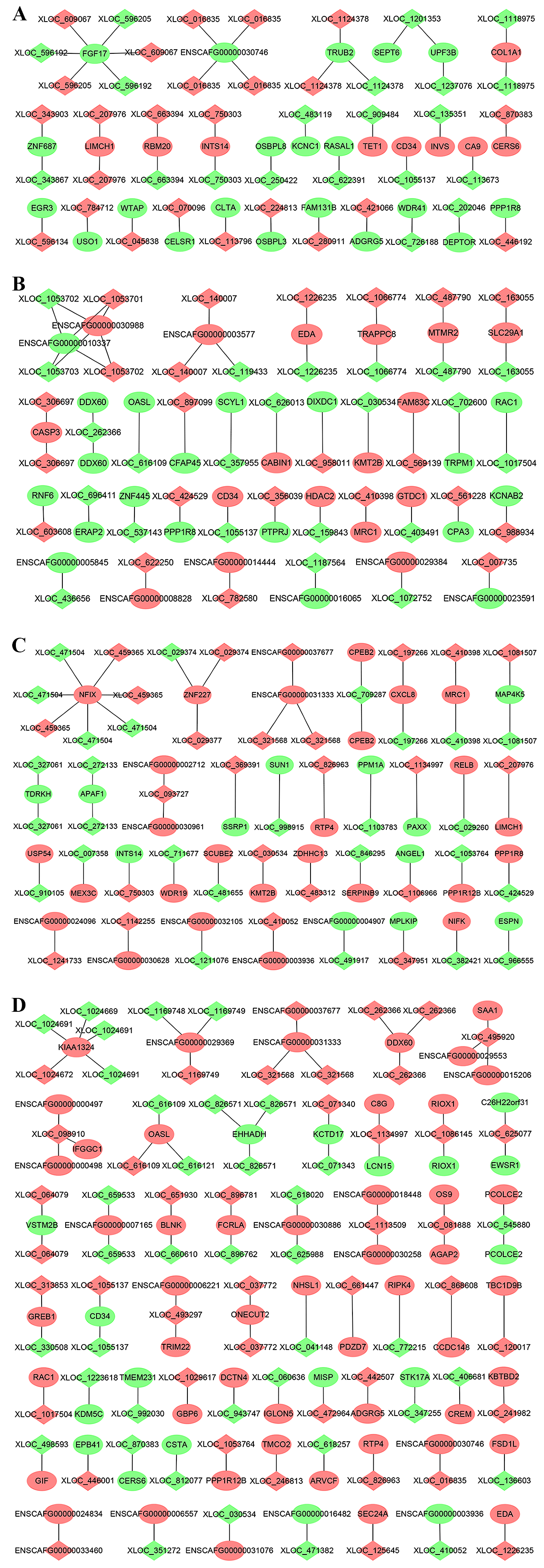

Supplement: Supplementary Figure 1 — Co-localization between the differentially expressed lncRNAs and their predicted differentially expressed target mRNAs. Red and green colors represent upregulated and downregulated transcripts, respectively. The ellipses denote mRNAs, and diamonds denote lncRNAs. (A–D) Represent the co-localization relationships at 12 hpi, 24 hpi, 96 hpi, and 36 dpi, respectively. [file Image_1.TIF]

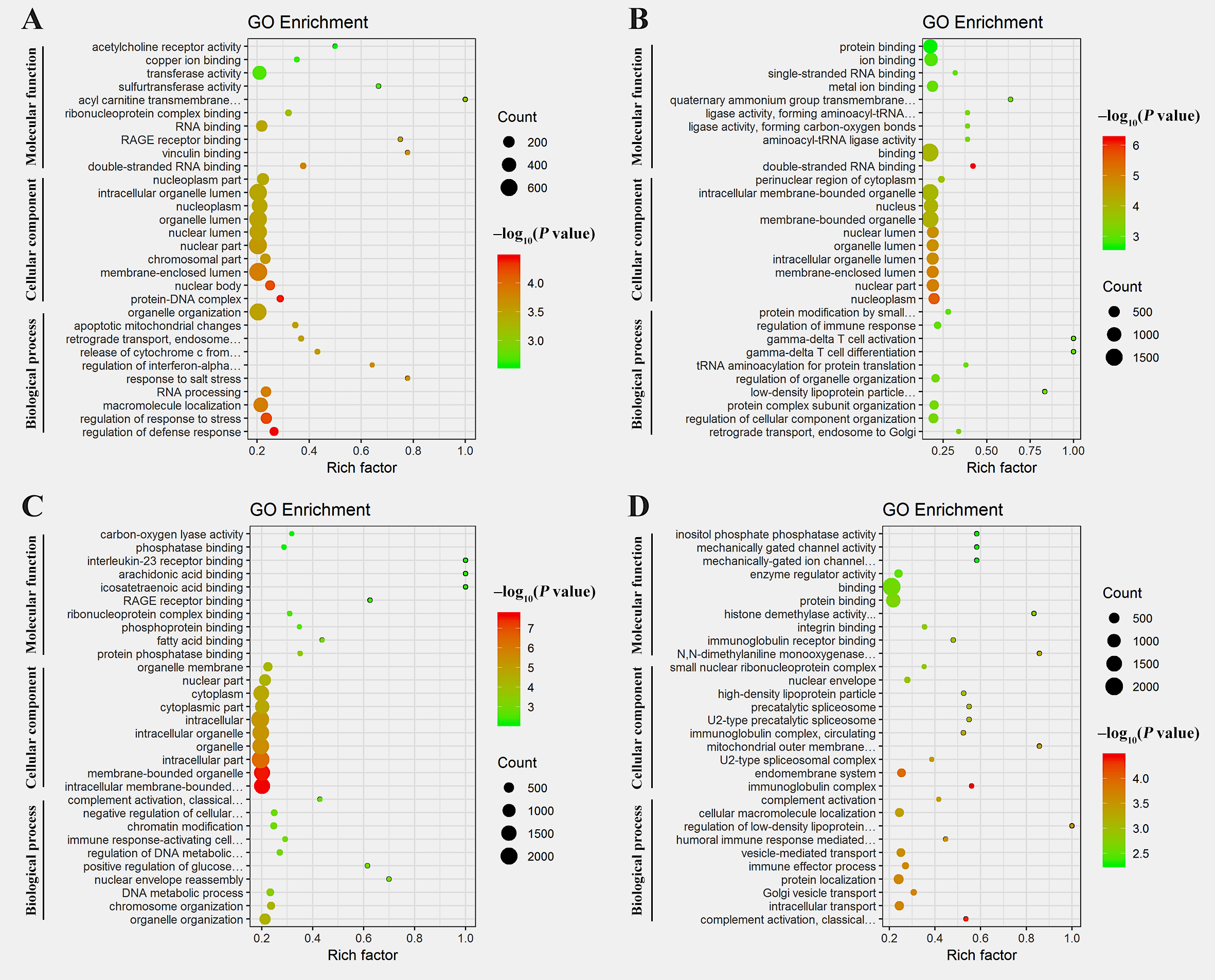

Supplement: Supplementary Figure 2 — Scatter plots of the top 30 enriched Gene Ontology (GO) terms (including biological process, cellular component, and molecular function categories) of the DElncRNA target genes at (A) 12 hpi, (B) 24 hpi, (C) 96 hpi, and (D) 36 dpi. The X-axis label represents the rich factor; the Y-axis label shows the GO terms. The rich factor reflects the proportion of DEmRNAs in a given GO term. The higher the rich factor, the greater the degree of enrichment. The color of the dots represents the enrichment. Score [–log10(P-value)], where red color indicates high enrichment, while green color indicates low enrichment. Dot size represents the number of DEmRNAs in the corresponding GO term (bigger dots indicate larger DEmRNA numbers). [file Image_2.TIF]

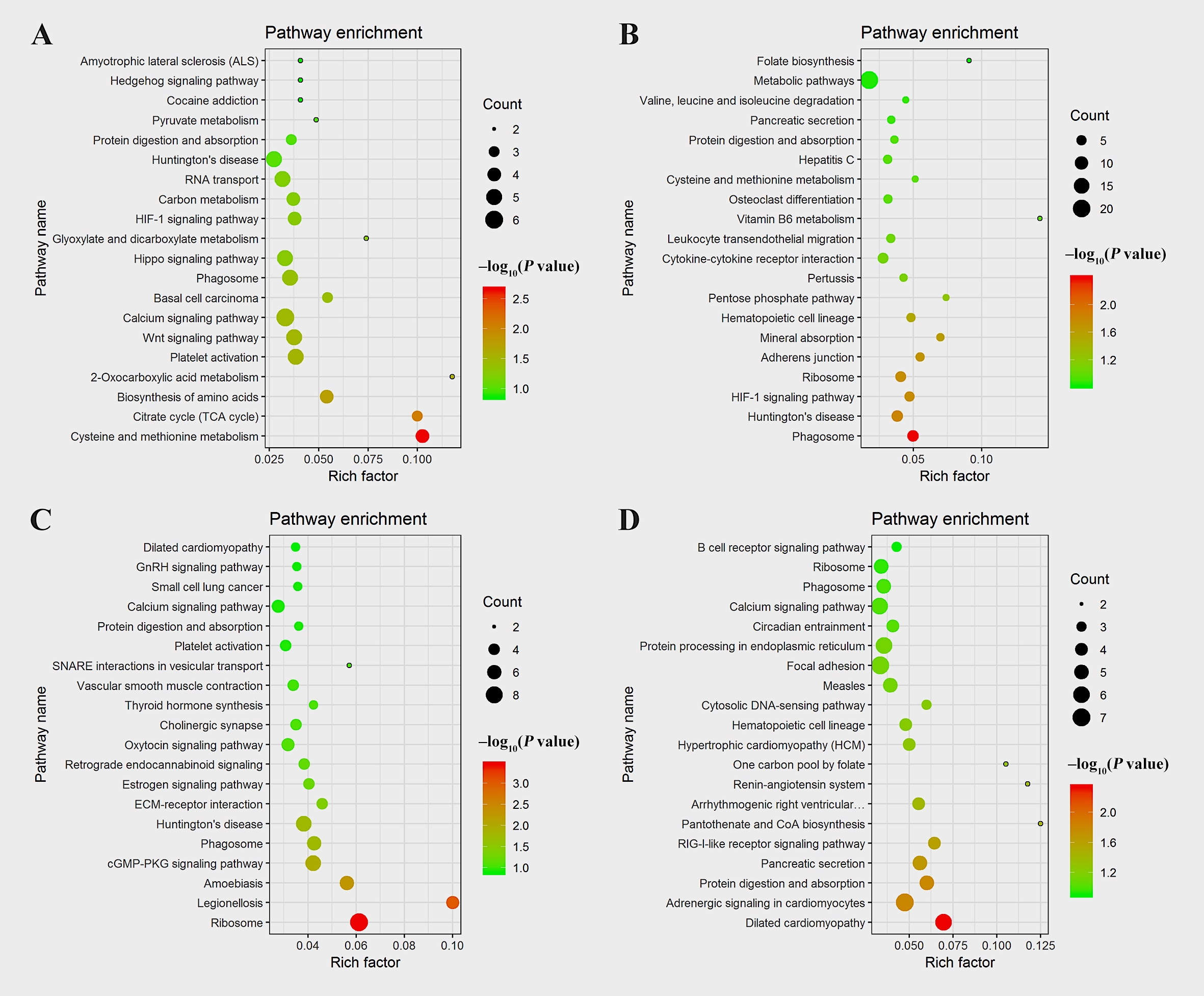

Supplement: Supplementary Figure 3 — Scatter plots of the top 20 enriched Kyoto Encyclopedia of Genes and Genomes (KEGG) pathways of the DEmRNAs at (A) 12 hpi, (B) 24 hpi, (C) 96 hpi, and (D) 36 dpi. The X-axis label represents the rich factor; the Y-axis label shows the KEGG pathways. The rich factor reflects the proportion of DEmRNAs in a given pathway. The greater the rich factor, the greater the degree of pathway enrichment. The color of the dots represents the enrichment score [–log10(P-value)], where red color indicates high enrichment, while green color indicates low enrichment. Dot size represents the number of DEmRNAs in the corresponding pathway (bigger dots indicate larger DEmRNA numbers). [file Image_3.TIF]
